# Molecular adaptation of telomere associated genes in mammals

**DOI:** 10.1186/1471-2148-13-251

**Published:** 2013-11-15

**Authors:** Claire C Morgan, Ann M Mc Cartney, Mark TA Donoghue, Noeleen B Loughran, Charles Spillane, Emma C Teeling, Mary J O’Connell

**Affiliations:** 1 Bioinformatics and Molecular Evolution Group, School of Biotechnology, Dublin City University, Glasnevin, Dublin 9, Ireland; 2 Centre for Scientific Computing & Complex Systems Modelling (SCI-SYM), Dublin City University, Glasnevin, Dublin 9, Ireland; 3 Genetics and Biotechnology Lab, Plant and AgriBiosciences Research Centre, National University of Ireland Galway (NUIG), Aras de Brun C306, Galway, Ireland; 4 Cold Spring Harbor Laboratory, Cold Spring Harbor, NY 11724, USA; 5 Program in Molecular Structure and Function, Hospital for Sick Children, Toronto, Canada and Departments of Biochemistry and Molecular Genetics, University of Toronto, Toronto, Canada; 6 School of Biology and Environmental Science & UCD Conway Institute of Biomolecular and Biomedical Research, University College Dublin, Belfield, Dublin 4, Ireland; 7 Current address: Department of Organismic and Evolutionary Biology and Museum of Comparative Zoology, Harvard University, 26 Oxford Street, Cambridge, MA 02138, USA

**Keywords:** Positive selection, Mammal molecular evolution, Telomere, Senescence, Longevity

## Abstract

**Background:**

Placental mammals display a huge range of life history traits, including size, longevity, metabolic rate and germ line generation time. Although a number of general trends have been proposed between these traits, there are exceptions that warrant further investigation. Species such as naked mole rat, human and certain bat species all exhibit extreme longevity with respect to body size. It has long been established that telomeres and telomere maintenance have a clear role in ageing but it has not yet been established whether there is evidence for adaptation in telomere maintenance proteins that could account for increased longevity in these species.

**Results:**

Here we carry out a molecular investigation of selective pressure variation, specifically focusing on telomere associated genes across placental mammals. In general we observe a large number of instances of positive selection acting on telomere genes. Although these signatures of selection overall are not significantly correlated with either longevity or body size we do identify positive selection in the microbat species *Myotis lucifugus* in functionally important regions of the telomere maintenance genes *DKC1* and *TERT*, and in naked mole rat in the DNA repair gene *BRCA1*.

**Conclusion:**

These results demonstrate the multifarious selective pressures acting across the mammal phylogeny driving lineage-specific adaptations of telomere associated genes. Our results show that regardless of the longevity of a species, these proteins have evolved under positive selection thereby removing increased longevity as the single selective force driving this rapid rate of evolution. However, evidence of molecular adaptations specific to naked mole rat and *Myotis lucifugus* highlight functionally significant regions in genes that may alter the way in which telomeres are regulated and maintained in these longer-lived species.

## Background

**Figure 1:**
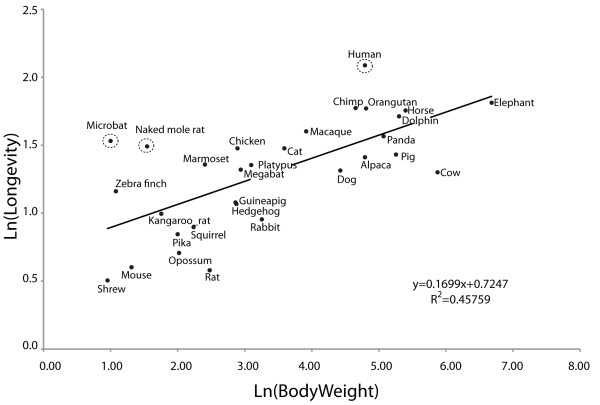
**Plot of the maximum longevity versus body weight for a collection of amniotes.** The natural log (lnL) of body weight is plotted against the lnL of longevity for the species analysed in this study. The regression line indicates a correlation between both life history traits. The exceptions are microbat (*M. lucifugus)*, human and naked mole rat circled to highlight their extreme values.

Placental mammals display a great deal of variation in many life history traits including maximum longevity and body mass. A correlation between certain life history traits has been observed with larger mammals tending towards longer life spans [1], Figure 1. Certain species do not adhere to this general rule and exhibit extreme longevity with respect to their body size. The naked mole rat and the microbat *Myotis lucifugu*s have a maximum longevity of 31 years and 34 years, but with small body sizes of 35 g and 10 g respectively [2] they are out of line with their expected longevity, Figure 1. In addition to this, both species do not exhibit the same age related deterioration such as hearing loss or reduced reproductive capabilities that are associated with human senescence [3,4]. For these reasons, both naked mole rat and microbat (*Myotis lucifugu*s) have been proposed as candidate study species to further our understanding of the mechanisms underpinning increased longevity and senescence [3,5]. Naked mole rats, microbats and human have all evolved increased life-spans but the underlying selective pressures leading to this extreme life history trait are most likely complex and varied.

Since the mid 1950s the role of natural selection in senescence has been debated [6-8] as reviewed by Kirkwood and Austad [9], and there is agreement that variation in longevity is potentially in response to predation risk [5]. Microbats have a niche advantage in that flight has reduced the range of predators possible [10]. This reduced risk of predation is thought to be a major contributing factor in the evolution of extended life-span. The naked mole rat lives in a subterranean habitat and is therefore largely protected from predation [5] but they are also a eusocial mammal. It is now recognised that social effects influence longevity and considering longevity in a kin-selection framework this places the eusocial naked mole rat with a strong selective pressure for increased longevity in a niche with reduced predation risk. In humans it has been proposed that increased life span is the result of selective pressure to provide care for dependents to increase their fitness (for a review on the application of kin selection theory to ageing/longevity theory see [11]). Indeed, even within species of anthropoid primates there is variation in longevity depending on the levels of care provided by the male or female, e.g. in species where the female provides the majority of care they have longer lifespans in comparison to males [12]. The 'disposable-soma’ theory of ageing predicts that reducing the extrinsic mortality risk, e.g. due to reducing predation, means a species can make substantial investments in growth and 'somatic maintenance’ as they will have more opportunities to reproduce, this is in contrast to a species with high mortality risk, that typically invest more in early reproduction than maintenance [8,9]. Species with low extrinsic mortality risk should also select against late onset deleterious mutations and these should therefore not accumulate with age [5]. Likewise, antagonistic pleiotropy caused by mutations that are beneficial early in life but have negative benefits later in life should not have the same effects in species with low mortality risks [13]. Selective pressures to improve the fitness of the next generation in reduced predation environments can result in increased longevity by adaptation of a variety of genes. Studying species with varying extrinsic mortality risks and divergent life spans should enable a better understanding of the molecular processes involved in ageing.

In 2009, Blackburn, Grieder and Stostak won the Nobel Prize for discovering that repetitive elements on the end of chromosomes (telomeres) maintain chromosome integrity and that the enzyme telomerase maintains these telomeres. In linear chromosomes, telomeres are crucial “TTAGGG(n)” repeat structures that protect the tips of chromosomes in both somatic and germ line cells. Telomerase along with a suite of DNA repair, telomere binding and chromatin regulators (collectively referred to as “telomere associated genes” throughout this paper), are key regulators of these protective caps, see review [14]. The telomere repeat sequence “TTAGGG(n)”, while conserved across vertebrates, can vary in length, for example humans have ~10-15 kb of telomere sequence at the tips of their chromosomes, while mice have approximately ~20-50 kb of telomere sequence [15,16]. Telomeres typically shorten during each cell division in the absence of telomerase expression, eventually reaching a critical length, which triggers cell death. This process is known as 'replicative senescence’ [17]. Maintenance of telomere length directly impacts the regenerative capacity of stem cells, and has been associated with premature ageing diseases such as Werner’s syndrome, Ataxia telangiectasia and Dyskeratosis congenita [18-20]. However, recent debate has centered on whether telomere length is correlated with body size rather than life span thereby calling into question the direct role of telomere length in chronological ageing [5]. Here we sought to determine if there were lineage-specific patterns of evolution in the proteins responsible for telomere length and maintenance unique to those lineages with extended longevity.

Adaptation at the molecular level is estimated by the ratio of non-synonymous substitutions per non-synonymous site (Dn) to synonymous substitutions per synonymous sites (Ds), referred to as ω throughout. An ω value of < 1 indicates that a gene is undergoing purifying selection, an ω value = 1 is indicative of genetic drift or neutral evolution, while ω > 1 signifies positive selection. The relationship between positive selection and protein functional shift has been validated biochemically through the rational mutagenesis of various fungal, plant and mammal enzymes [21-24]. Whilst positive selection has been reported for between 38% and 62% of protein coding genes in mammals [25], it is generally held that the majority of codon positions are evolving neutrally or under purifying selection [26].

Signatures for adaptive evolution have been shown for regulatory regions and protein-coding genes [27,28]. In the case of protein coding regions the genes implicated are frequently those that have emerged through gene duplication [29]. Gene duplication is a well-known source for new gene function [30] although the precise mechanism of neofunctionalization remains debated [31,32]. In general it is observed that most duplicates tend to become subfunctionalized or pseudogenized in the absence of purifying selection [32]. Recombination can introduce variability to populations [33-35] and can also influence the process of natural selection [36]. Non-adaptive evolutionary forces (such as recombination, the accumulation of deleterious mutations and the variation in demographic history across these species) are also major contributing factors in shaping gene evolution [37] and should also be considered.

Here we assess whether there is enrichment for positive selection in lineages with increased longevity, i.e. the naked mole rat, *M. lucifugus* and human in those genes responsible for telomere maintenance and integrity. We determine species-specific patterns of adaptive evolution in microbat, naked mole rat, and human and we explore the molecular adaptations that have occurred uniquely in the human lineage using population level data.

## Results and discussion

The 56 telomere associated genes for this study [14] were clustered into 54 gene families across 26 placental mammals and 4 outgroup species (*Monodelphis domestica, Ornithorhynchus anatinus, Taeniopygia guttata* and *Gallus gallus*). Multiple sequence alignments (MSAs) were generated using both distance and evolutionary aware methods [38,39] ensuring a comprehensive exploration of alignment space. Sequences with less than 60% coverage over the entire length of the MSA, or individual columns that did not have 60% minimum coverage across a position, were removed using trimAl [40], giving a final dataset of 52 gene family alignments for further analyses. These gene families were composed of 4 multigene families, 14 families that contained lineage-specific gene duplications and 34 single gene orthologous families. All MSAs were of good quality (norMD > 0.6) [41], ranging between 786 bp and 5514 bp in length and contained between 14 and 48 species. The profile of the dataset is described in Additional file 1.

### Adaptive and non-adaptive mechanisms shape the evolution of telomere associated genes

Codon models of evolution were applied to the 52 gene families using CodeML [42,43]. The CladeC model [44,45] allows for a labeled branch or clade to have different selective pressure acting in the foreground and the background, thus providing a framework for the subtle detection of divergent evolution among clades. The CladeC model was compared against the m2_rel model [46] and significance was assessed by comparing twice the difference in likelihood scores (2ΔlnL) to the chi-squared table based on the degrees of freedom between the two models. We controlled for false discovery rates using the Benjamini-Hochberg procedure [47] and we adjusted the P-values accordingly. Using this procedure the LRTs between CladeC v m2_rel, modelA v modelAnull, and modelA v m1Neutral were deemed significant if the adjusted P value (p-adj) < 0.01, see Additional file 2. The branch-site modelA [48] allows for positive selection to occur in the labeled foreground branch while on all other branches (background), positive selection is not permitted. ModelA is compared to null models m1neutral and modelAnull, and significance is assessed using the same method as described for the CladeC model. The posterior probability (PP) of positively selected sites was estimated using the Bayes Empirical Bayes (BEB) method [48]. To minimize the detection of false positives only sites where a PP > 0.90 are listed (see Additional file 3). By applying both model types to our data we can (i) assess the heterogeneity of divergent evolution across clades, and (ii) obtain a profile of the proportion of species that have undergone positive selection in their telomere associated genes.

**Figure 2:**
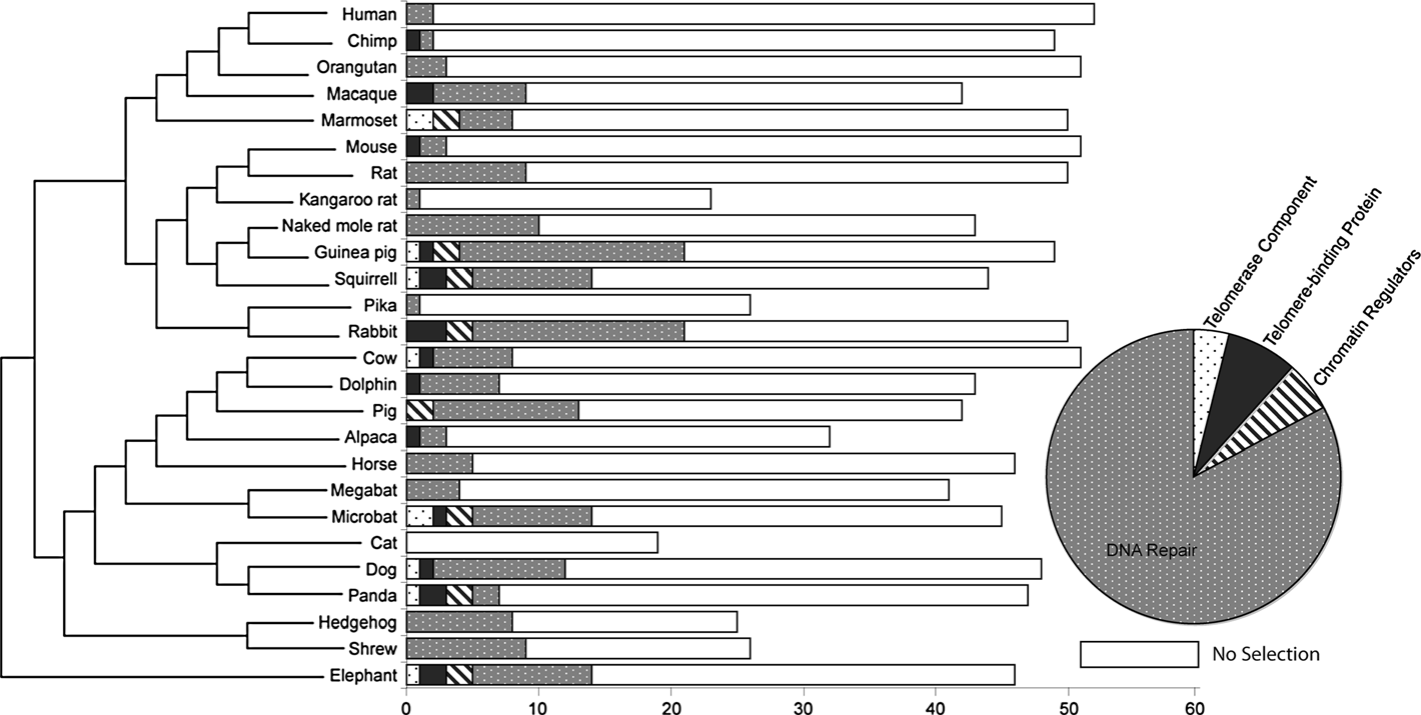
**Overview of levels of lineage-specific positive selection on telomere associated genes in each lineage of mammal.** Each of the 52 telomere associated genes are categorized into 4 major functional groups (colour coded as per pie chart inset). On the left is the placental mammal phylogeny used. The maximum length of each horizontal bar in the histogram depicts the overall number of times that species was represented in the total dataset of 52 gene families. The shaded portions of the bars correspond to the functional categories (as per colour scheme in the pie chart), the size of these shaded regions represents the proportion of genes in that category that are identified as having undergone lineage-specific positive selection.

The CladeC model indicates a large proportion of lineages in the analysis have undergone divergent evolution with 19/52 genes calculated has having between 1 and 3 branches diverging with an ω value > 1, while the remaining branches are diverging with ω < or = 1. This result highlights the heterogeneity in divergence rates across lineages. We then tested our data using lineage-site Model A and identified positive selection in 24/26 placental mammals across 48/52 of the gene families tested, for summary of results see Figure 2 and Additional files 2 and 3. The effect of saturation at the synonymous level (Ds) on ω estimates has been debated in the literature [49-51]. Recent studies have shown that Ds saturation is more likely to contribute to loss of power in the analysis rather than increased false positive discovery [51]. We have estimated the Ds level across our data using the Yang and Nielsen (2000) method in the PAML v. 4.4 [42] and found 15 genes with evidence of saturation at silent sites. These 15 genes are therefore likely to have reduced power but they have been retained in all subsequent analyses as they are unlikely to produce false positive results. Trees estimated from Ds have been made available in Additional file 4. The mean percentage of genes with signatures of positive selection was estimated at 15.24%±11.11. There were outliers such as the guinea pig where a large proportion (38.78%) of the homologs from this lineage displayed signatures of positive selection, on the other hand there were lineages such as cat and pika where none of the homologs had evidence of positive selection.

We tested the hypothesis that increased longevity, body mass, or the ratio of longevity to body mass were correlated with increased levels of positive selection using a Pearson correlation. Comparisons of the percentage of genes under selection across mammals showed weak negative correlation at the 10% significance level when compared to longevity (cor = -0.3297, p-value = 0.09997) and no correlation when compared to body mass (cor = 0.1036, p-value = 0.6144) or longevity to body mass ratio (cor = 0.1536, p-value = 0.4537). To correct for the influence of gene length on our results, and rather than simply comparing the number of sites to the life history traits of interest, we also compared the life history traits with the proportion of sites under positive selection as a function of gene length. The results were similar to those obtained using the raw positively selected gene counts: longevity (cor = -0.3453, p-value = 0.0840), body mass (cor = 0.0031, p-value = 0.988), and the longevity to body mass ratio (cor = -0.0227, p-value = 0.9125).

Non-adaptive processes such as recombination have been shown to dramatically influence the evolutionary trajectory of a protein sequence [52], but they can also create signatures that mimic positive selection. Recombination events were detected in 47/52 of genes in the dataset, and in all lineages. It is difficult to tease apart signatures for positive selection from recombination as accurate break point detection in recombination analysis is difficult [53], and these processes are not mutually exclusive with positive selection known to occur within recombinant regions [54]. Therefore, the genes with evidence of recombination were not discarded from the analysis, they were analysed for selective pressure variation and the positions of recombined and positively selected sites were compared. It is clear that the interactions between adaptive and non-adaptive events are complex and further development of methods is required to adequately tease apart the effects of these evolutionary processes [55]. We have reported our findings in Additional file 5 but cannot discount the possibility that both processes are acting on these genes simultaneously.

### Naked mole rat-specific positive selection

**Figure 3:**
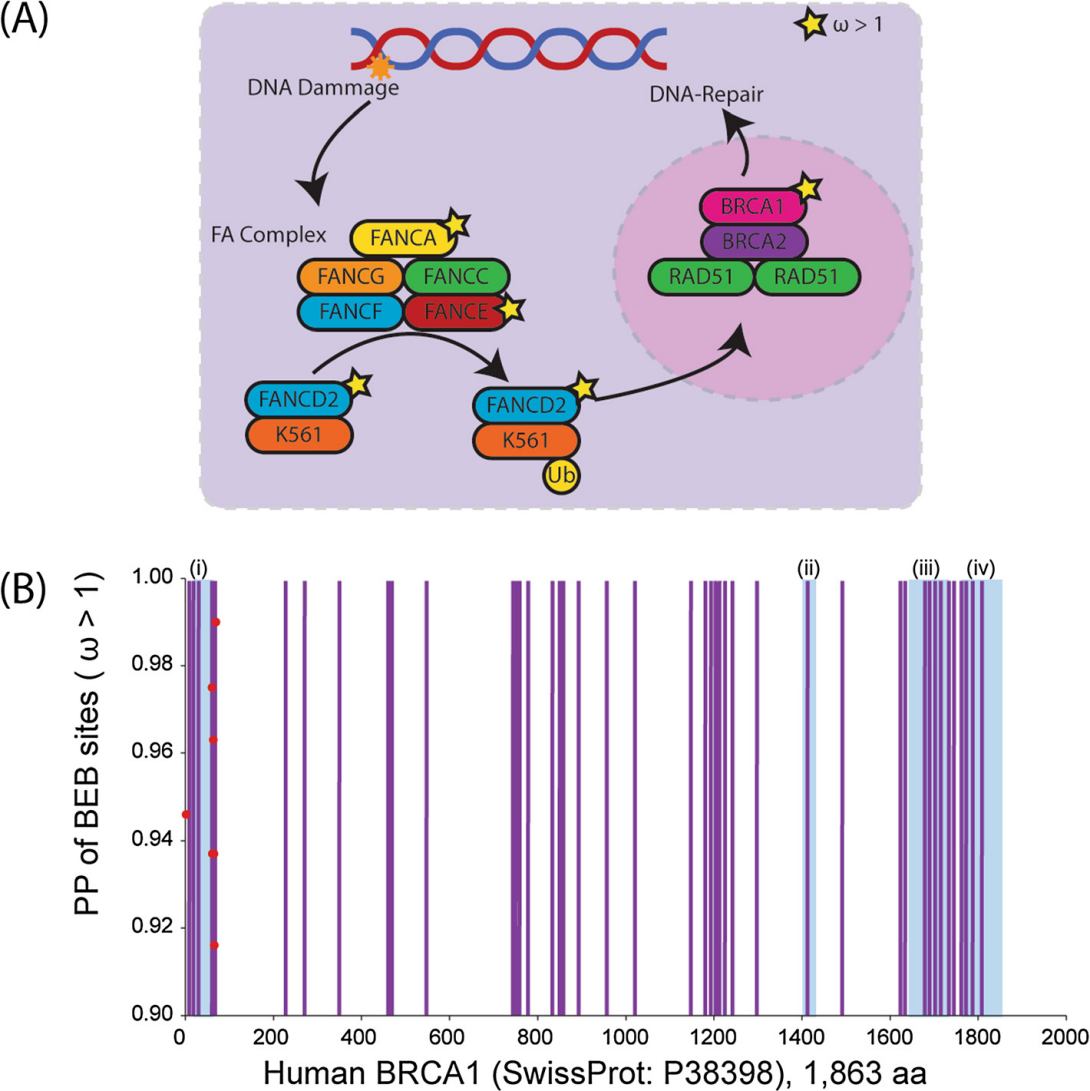
**Lineage-specific positive selection in the naked mole rat BRCA1/FANC pathway. (A)** The BRCA1/FANC pathway with genes under positive selection in naked mole rat denoted by a star. **(B)** Is a graphic of the BRCA1 protein from position 1 to 1853 on the x-axis. Positively selected sites (red dots) from naked mole rat were mapped to model sequence (from human). The posterior probability for each site is given on the y-axis. Functional information extracted from the human entry in swissport is colour coded as follows: blue regions represent (i) Zinc Finger, (ii) Interaction region with PALB2, (iii) BRCT1 domain, and (iv) BRCT2 domain while purple vertical lines are cancer causing natural variants.

We tested 43 gene families where the naked mole rat lineage was labeled as foreground, 3 of these were significant for differential divergence rates under the CladeC model, with 1 of these genes (*RAD51D*) evolving under positive selection. Lineage-specific selective pressure analysis under modelA identified 9 genes under positive selection: *ANKD17*, *ABL1*, *FANCE*, *MSH3*, *FANCA*, *SLX4*, *BRAC1, XRCC5* and *FANCL*, none of which overlap with positively selected genes in *M. lucifugus* or human. Interestingly, all of the positively selected genes were involved in DNA repair. Three of these positively selected genes: *BRCA1, FANCE,* and *FANCA*, work together in response to DNA damage in the Fanconi anaemia/BRCA pathway (Figure 3A) and *BRCA1* has been shown to play a crucial role in telomere maintenance [56]. We mapped the positively selected sites from naked mole rat for *BRCA1* to amino acid positions in the human BRCA1 ortholog to extract functional information (Figure 3B). The seven positively selected sites occur in close proximity to one another, all are located within the Zinc Finger domain - crucial for protein-protein interactions [57], and all are adjacent to natural variants associated with cancer in humans. In the naked mole rate lineage there was no positive selection identified in genes involved in telomere-binding and insufficient sequence data was available to test the telomerase components.

### Microbat (*M. lucifugus)*-specific positive selection

In total there were 45 sequence alignments with *M. lucifugus* representation, these were analysed using the CladeC model and 2 showed evidence of differential divergence in the microbat lineage. The first of these two genes, *ANKHD1,* did not show evidence of positive selection. However, *DKC1* was identified as being positively selected and with an ω value of 1.92. The DCK1 protein is a component of the telomerase enzyme that directly maintains telomere length [58]. Missense mutations in this gene result in X-linked dyskeratosis congenital (XDKC) [59], a congenital disorder that causes pre-mature ageing. There were no individual sites using the BEB calculation that had a PP > 0.90 of being positively selected under the CladeC model, therefore we applied modelA to gain more site-specific information.

**Figure 4:**
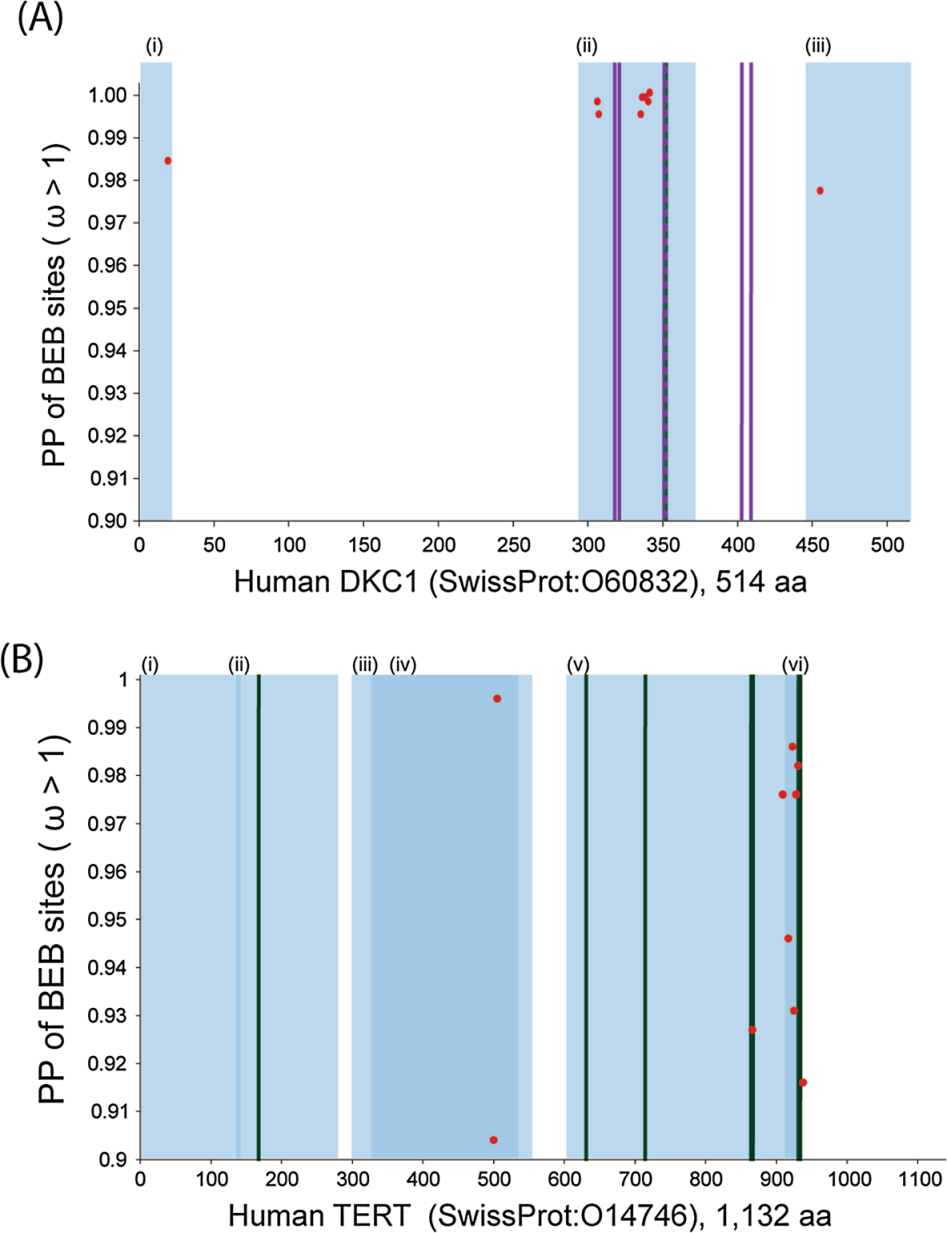
**Functional assessment of positively selected sites from *Myotis lucifugus *by comparison to human orthologs.** In both **(A)** and **(B)** the amino acid position of the human gene is given on the x-axis and the PP of a given site being under positive selection is given on the y-axis. **(A)** are the results for DKC1 and **(B)** are the results for TERT. Positively selected sites are shown as red dots throughout and different domains are denoted with roman numerals and are highlighted by blue semi-transparent blocks. For **(A)** blue shaded regions are: (i) nucleolar localization, (ii) PUA domain, and (iii), nuclear and nucleolar localization respectively. In **(B)** mutagenesis sites which result in reduced telomerase activity are represented by vertical green lines, and blue shaded regions are: (i) RNA-interacting domain 1, (ii) Required for regulating specificity for telomeric DNA, (iii) Required for oligomerization, (iv) RNA-interacting domain 2, (v) Reverse transcriptase, and (vi), Required for oligomerization. The purple vertical lines in **(A)** represent the natural variants resulting in XDKC, and the vertical green lines in **(B)** represent the position of the natural variants resulting in HH0053.

Within the *M. lucifugus* lineage, modelA identified 10/45 gene families as evolving under positive selection: *ANKHD1, RBL1, TNKS, DKC1, NBN, EXO1, BRCA2, BRIP1, SUV39H1, TERT*. Previous analyses have proposed the *BRCA2* gene as under positive selection in the *Myotis davidii* species [60]. To determine the functional importance of positive selection on these genes we mapped the *M. lucifugus* positively selected sites in *DKC1* and *TERT* to the orthologous positions in the human - Swiss-Prot sequences O60832 and O14746 respectively, see Figure 4. The majority of positively selected sites (8/10 sites) in the *M. lucifugus* lineage for the *DKC1* gene map to the PUA (pseudouridine synthase and archaeosine transglycosylase) domain involved in RNA modification [61]. Within this region, natural variants associated with XDKC are also found, this is a disorder involving defective telomere maintenance [62] (Figure 4A). In the *M. lucifugus TERT* gene, there were 11 sites identified as being positively selected (PP > 0.90, BEB estimates). This gene has been well characterized and we were able to map these positively selected sites to functionally important regions. As shown in Figure 4B, region (iii) and (iv) of the TERT protein are required for oligomerization and RNA-interacting domain 2, essential for interacting with another telomerease component (TERC) and for DNA synthesis. Within this region we identified two sites under positive selection. The remaining positively selected sites were identified in either region (v) or (vi) of the TERT protein both of which are critical regions in telomere maintenance as they are required for reverse transcriptase and oligomerization activity respectively. Mutagenesis studies have been carried out on the human *TERT* to identify regions that result in reduced telomerase activity, sites implicated are: D868A, D869A and positions 930–934 [63,64], and these fall within close proximity to the sites identified in *M. lucifugus* as positively selected. This provides us with strong evidence that this 'hot spot’ of positive selection in *M. lucifugus TERT* may have tangible functional impact on altered telomere maintenance in this species.

### Human-specific positive selection

Two genes were identified as being positively selected in the human population using modelA with p-adj < 0.01. To address whether the signatures of divergent evolution are manifest within modern human populations we examined the pattern of segregation of single nucleotide polymorphisms (SNPs) occurring in all genes that were predicted to have signatures of positive selection in the human lineage. We used HapMap data for East Asian (A), Northern and Western European (C), and African Yoruba (Y) populations and estimated the integrated haplotype score (iHS) [65] for each population using the SNP@Evolution database [66]. The iHS is a measure of segregation of an allele within a population, iHS scores > +2 indicates that the allele is segregating in the population (under positive selection) while an iHS score < -2 indicates that the allele is reducing in frequency [65].

Using the modelA suite of LRTs we identified *MRE11A*, and *ERCC1* as having signatures of positive selection (Table 1), but there were no SNP data available for these two genes and therefore a population level analysis was not possible. We found that the *WRN* gene displayed evidence of positive selection (ω = 1.39) with a p-adj = 0.026. Although this p-adj value is higher than previously set, we believe that the higher quality data for human merits further exploration of this gene using available SNP data, the results are described below.

**Table 1:** Results for the analysis of telomere associated genes using modelA

**ModelA**	**Human**	***Myotis lucifugus *** **(Microbat)**	**Naked mole rat**
Positive selection predicted (ω > 1)	MRE11A, ERCC1^+^	ANKHD1, RBL1^+^, TNKS^+^, DKC1, NBN^+^, EXO1^+^, BRCA2, BRIP1, SUV39H1^+^, TERT^+^	ANKRD17, ABL1^+^, FANCE, MSH3, FANCA, SLX4, BRCA1^+^, FANCL, XRCC5
Lineage-specific positive selection | families tested	2 | 52	10 | 45	9 | 43

The results summarized here are only those that were significant following LRT analyses of modelA versus m1Neutral model, and modelAnull for human, naked mole rat and *M. lucifugus*. The names of the genes identified in each species as having undergone positive selection are given (where ^+^denotes Ds saturation present in alignment). The number of gene families identified as under positive selection in each species is represented along with the total number of families tested for that species.

Under the CladeC model we identified one gene that showed evidence of differential divergence in the human lineage with p-adj < 0.01, *RAD51D* (Table 2). Examining a more relaxed cut-off criteria of p-adj < 0.10, both *WRN* (ω = 2.33, p-adj = 0.068) and *RBL1* (ω = 1.52, p-adj = 0.068) displayed evidence of positive selection for the species-level comparison under with the CladeC model. We performed further analysis on *RBL1* and *WRN* to determine whether these genes are under ongoing positive selection or whether these sites are fixed in all modern human populations.

**Table 2:** **Summary of results of codon models of evolution for the 3 mammals with greatest longevity to body mass variation, i.e. human, naked mole rat and *Myotis lucifugus***

**CladeC**	**Human**	***Myotis lucifugus *** **(Microbat)**	**Naked mole rat**
ω > 1	NA	DKC1	RAD51D
Lineage-specific positive selection | families tested	0 | 52	1 | 45	1 | 43
ω < 1	RAD51D	ANKHD1	ANKRD17 and BRCA2
Lineage-specific divergence | families tested	1 | 52	1 | 45	2 |43

The significant CladeC versus M2_rel model LRT analysis are summarised. Results for both positive selection (ω > 1) and divergent purifying selection (ω < 1) are shown for the three species of interest.

**Figure 5:**
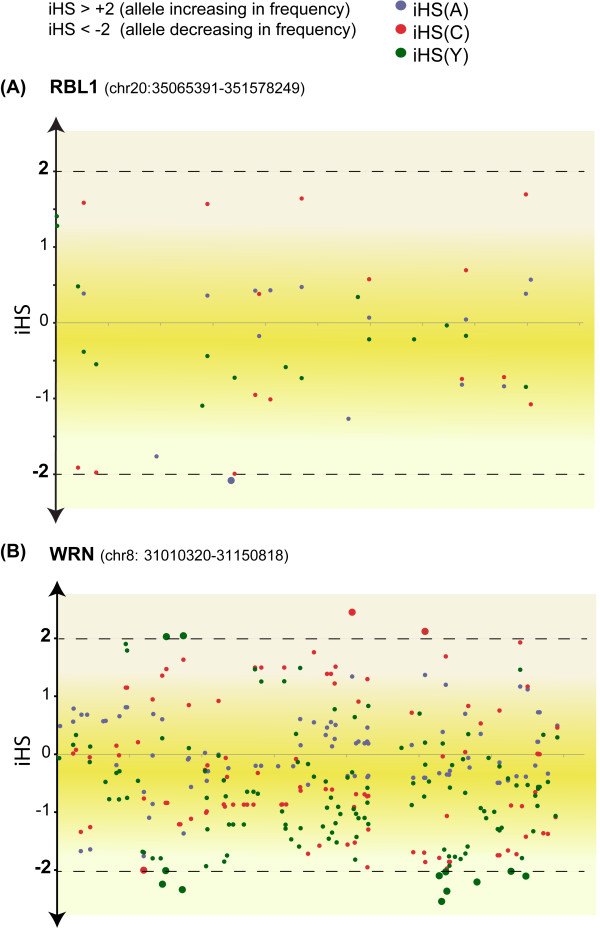
**An overview of SNP frequency within human populations.** The results for iHS analysis of the RBL1 gene is given in panel **(A)** and for the WRN gene id given in panel **(B)**. The iHS scores for SNPs within the East Asian (A), Northern and Western European (C), and African Yoruba (Y) populations are shown as blue, red and green dots respectively. The position of SNPs on the x-axis is proportional to the distance from the first SNP within the gene.

The *WRN* gene is associated with a disorder of premature ageing called Werner syndrome [67] and it showed evidence for positive selection under both lineage specific suites of LRTs applied in this study. The population level analysis revealed that *WRN* had an iHS > +2, indicating a continued positive selective pressure acting on this gene in modern humans with two independently segregating alleles in both the European (C) and African Yoruba (Y) populations (Figure 5). RBL1 was analysed in a similar way but showed no evidence of ongoing positive selective pressure (Figure 5).

In total nine genes were estimated to have signatures of divergent evolution under the CladeC model, on analysis of the human population level data five showed significant levels of segregation (positive selection), Table 3 shows a summary of the number of associated SNPs in each case. These results indicate that there are detectable levels of positive and negative selection acting within modern human populations for genes involved in telomere maintenance. These genes have fundamental roles in key processes that maintain genome integrity, i.e. DNA repair (*RAD51D* and *WRN*) and chromatin regulation (*RBL1*).

**Table 3:** Measures of ongoing selective pressures in human populations for a subset of telomere associated genes

**Gene name and significance level**	**iHS**	**A**	**C**	**Y**	**Total SNPs**
**ATM**^**@5%**^	**> +2**	**3**	**5**	**0**	**158**
	**< -2**	**0**	**3**	**18**	
**BRCA1**^**@5%**^	**> +2**	**0**	**1**	**0**	**108**
	**< -2**	**0**	**0**	**1**	
**WRN**^**@10%**^	**> +2**	**0**	**2**	**2**	**306**
	**< -2**	**0**	**1**	**10**	
**RBL1**^**@10%**^	**> +2**	**0**	**0**	**0**	**88**
	**< -2**	**1**	**0**	**0**	
**ANKHD1**^**@5%**^	**> +2**	**0**	**0**	**0**	**49**
	**< -2**	**0**	**1**	**1**	

Each gene identified as diverging within the ancestral human population at p-adj (0.05) and p-adj (0.10) significance levels under the CladeC model are listed with the number of SNPs with iHS > 2 and the number of SNPs with an iHS < -2 for the East Asian (A), northern and Western European (C), and African Yoruba (Y) populations. The total number of SNPs known for each gene is given in the final column.

## Conclusion

There have been many competing hypotheses proposed to explain the observed variation in longevity and ageing in animals, yet molecular evolutionary studies have been lacking. While clear correlations exist between life history traits such as longevity and body mass, there are a number of species that do not follow these general rules and these are particularly interesting for improving our fundamental understanding of the process of longevity and ageing. We sought to test if there were signatures of positive selection in genes associated with telomere maintenance that correlated with incidences of increased longevity and body size in mammals. We found a weak correlation between longevity and levels of positive selection and no correlation between the levels of positive selection and body mass. It was not possible for us to pinpoint one single life trait as the main contributor to the strong signature for positive selection in telomere associated genes that we observe across mammals. Our results instead are suggestive of a more complex selective force shaping the evolution of these genes that is likely to incorporate variation across species in demography, metabolic rate, germ line generation time, as well as body size and longevity. As telomere-associated genes underpin important functions in maintaining genomic integrity, cancer as a selective force, or more specifically the selective pressure to reduce the incidence of juvenile cancers, could contribute towards these strong signals for protein functional shift that we observe in this gene set thereby providing an alternative to direct selection for increased longevity.

We cannot completely discount any of the current competing hypotheses for increased longevity, nor can we discount the role that other genes outside of telomere maintenance may play in increased longevity. While we have applied rigorous statistical testing to our dataset, ω > 1 may not always be a indicator of protein functional shift as it may reflect variations in effective population size [68], recombination events [52], biased gene conversion [69] or relaxation of functional constraint [70]. To this end we also examined non-adaptive selective forces on this genes. The proteins in our dataset control very important cellular functions and one might expect to find signals of strong purifying selection, to the contrary we observe high levels of both non-adaptive and adaptive evolutionary events in the data. Identifying proteins under species-specific positive selection is biologically significant as it is synonymous with protein functional shift. Here we have identified positive selection in a large number of proteins and lineages with significance values set to p-adj < 0.01 (CladeC 19/52 proteins and 15/26 placental mammals, modelA 48/52 proteins and 24/26 placental mammals), but of particular interest are the naked mole rat (CladeC 1/43 and modelA 9/43), microbat (Clade C 1/45 and modelA 10/45) and human (CladeC 0/52 and modelA 2/52) lineages all of which manifest increased longevity. It is particularly interesting that there was no overlap in the specific genes identified as having signatures of positive selection in either of the lineage-specific analyses (CladeC or modelA), and these genes were non-overlapping between the naked mole rat, *M. lucifugus* and human. Despite sharing the life history trait of increased longevity, each of these lineages has different strategies for reproduction and survival that can contribute to these different selective regimes, e.g. in the eusocial system adopted by the naked mole rat it is the older females that produce the most offspring [71], the ecological niche of the *M. lucifugus* and naked mole rat reduce risk of predation [72], and the variation in effective population sizes across all three species results in very different levels of background mutation upon which natural selection can act.

As well as increased longevity, *M. lucifugus* and naked mole rat have evolved the ability to postpone senescence. We have identified signatures of positive selection acting within regions of the genes directly involved in telomere maintenance in *M. lucifugus* (*DKC1* and *TERT*) and within the Fanconi anaemia/BRCA pathway DNA repair pathway in naked mole rat. These signatures of adaptation could be the result of selective pressure for delayed onset of deleterious attributes of ageing. The human lineage represents a unique situation where there is an observed increased longevity but there is no postponement of the ageing process. The identification of two genes (*RBL1 and WRN*) under positive selection in the human lineage spurred us to examine modern human populations for signatures of ongoing adaptive evolution in these proteins. The observed pattern of segregation in the *WRN* gene in European and African Yoruba populations is suggestive of continued positive selective pressure in modern human populations on telomere maintenance processes. There are of course a number of competing selective pressures acting on human populations (as well as a bottle neck in population size that can contribute to fixation of slightly deleterious mutations), and while pathogen load has been proposed as the most dominant driver of adaptation in the human lineage [70], cancer selection has a distinct possibility as a contributing factor [69,73]. Indeed many of the sites identified in this study as positively selected are in close proximity to (or directly associated with) cancer.

As positive selection and protein functional shift are strongly correlated, identifying proteins under positive selection in specific lineages lends itself to more accurate molecular modeling of ageing, and cancer across species. Furthermore, the identification of specific molecular adaptations in telomere associated proteins in species with increased longevity, i.e. naked mole rat, human and *M. lucifugus*, provides us with an important fundamental step forward in our understanding of the diverse mechanisms involved in the evolution of increased longevity.

## Methods

### Data assembly

We obtained 29 completed vertebrate genomes through the Ensembl web server (http://www.ensembl.org/) [74]. Coding DNA sequences (CDSs) were also obtained for the naked mole rat genome from the database (http://mr.genomics.org.cn/page/species/index.jsp) [75]. For details on all 30 species genomes used in this study see Additional file 6. The 56 telomere associated genes were taken from Blasco [14]. A reciprocal mpiBLAST [76] was conducted at the amino acid level across all 30 genomes using the 56 genes as queries (e^-6^ for all genes). BLAST output files were clustered into families using orthoMCL [77] and 52 gene families were assembled.

### Alignment generation and alignment editing

Alignment is crucial for the detection of selective pressure variation in sequences, therefore we ensured the most statistically significant alignment was used for each protein family in the analysis. Multiple sequence alignments (MSAs) were generated for each gene family using two different alignment approaches, some with more than one alignment algorithm and compared the results. Firstly we applied “sequenced based” alignment methods using AQUA [39], this incorporated MUSCLE [78] and MAFFT [79] alignment packages, and a refinement algorithm RASCAL [80]. Secondly we applied the “phylogenetically aware” method PRANK with the '+F’ option to account for insertion deletion events [38]. The REFINER method [81] was used to assess the quality of all of the resulting alignments from the different algorithms using the estimated norMD values [41]. For each gene family, the alignment with the highest norMD score was used and where more than one alignment had an equal top score the alignment method was chosen at random. The distribution of the mean percentage identity (Mean %ID) was calculated for each alignment using trimAl [40]. Our criterion for inclusion in the analysis was that there must be at least 60% overlap with the entire alignment (by taxon and by site), this was to ensure that we had deep coverage of at least 60% of the protein. Regions that did not meet this strict criterion were removed using trimAl [40]. Further manual editing of the alignment was performed using Se-Al [82]. All final alignments had a norMD score > 0.6 and have been provided in Additional file 7.

### Phylogeny reconstruction

The most appropriate protein evolutionary model for each alignment was assessed using ModelGenerator v.85 [83]. Using the best-fit model the phylogenetic reconstruction was carried out using hybrid MrBayes v.3.1.2 h [84]. Two independent MCMC chains were run for 1.5 million generations and with samples every 10th run. The first 375,000 trees were discarded as burnin. Majority rule consensus trees were generated for each gene family from the distribution post-burnin. Gene trees for the single gene ortholog families were compared against the canonical species phylogeny [85,86] under the Shimodaira-Hasegawa (SH) [87] test in Tree-Puzzle v5.2 [88]. Phylogenetic trees have been provided in Additional file 8.

### Analysis of lineage-specific selective pressure variation

All extant lineages were tested for positive selection using lineage-site specific modelA and modelAnull models in CodeML [42,43,48] using four starting ω values (0, 1, 2 and 10). Both modelAnull and m1Neutral model were compared to modelA using a likelihood ratio test. The p-values were adjusted (p-adj) for multiple testing using the Benjamini-Hochberg procedure [47] and p-adj < 0.01 was taken as significant.

The CladeC model was applied to all extant branches to test for divergent evolution, and was compared to the M2_rel model described by [46]. As both models have been described as having uneven likelihood surfaces [46] the following ω starting values were applied; 0.001, 0.01, 0.1, 0.25, 0.5, 0.75, 1, 2, 3, 4, 5 and 10. The likelihood ratio test was used to determine if the CladeC model was a statistically better fit than the m2_rel model and if the adjusted p-values under the Benjamini-Hochberg procedure [47] were less than 0.01, the CladeC model was deemed significant [46].

Only sites that are found to have a posterior probability (PP) > = 0.90 using the BEB method are reported, therefore reducing the probability of false positives [48]. The complete set of Likelihood Ratio Test (LRT) statistics and selective pressure heterogeneity results are provided in Additional files 2 and 3.

### Test for saturation of synonymous substitutions (Ds)

The Yang and Nielsen (2000) method was implemented in PAML [42] to estimate synonymous and non-synonymous substitution rates between two sequences and assess whether the number of silent substitutions per silent site were saturated (as indicated by Ds > 2.0). Saturated Ds could reduce power to estimates of ω [89]. Results have been provided in Additional file 4.

### Correlation test

The percentage of genes in this dataset identified as having signatures of positive selection as well as the proportion of sites identified as being positively selected (value taken if modelA was significant) for the genes tested in each lineage were plotted against lineage longevity, body mass and the ratio of longevity to body mass. A Pearson correlation test was performed using R [90], significance was taken at the 5% level.

### Recombination detection

Recombination analysis was performed using RDP4 [91] and the following primary exploratory recombination signal detection methods were employed: RDP [92], GENECONV [93], BOOTSCAN/RESCAN [92], MaxChi [94], Chimaera [35], SiScan [95], 3Seq [96]. A secondary method, PhylPro [97] was used to assess the recombination results of the primary methods. The results are provided in Additional file 5.

### Human population level analysis

Integrated haplotype scores (iHS) [65] were obtained from SNP@Evolution [66] that incorporates HapMap release II source data in the following populations: East Asian (A), Northern and Western European (C), and African Yoruba (Y). An allele which receives an iHS score > +2 is deemed to be increasing in frequency in a population signifying continued segregation while an allele with an iHS score of < -2, indicates that the allele is decreasing in frequency.

## Competing interest

The authors declare that they have no competing interest.

## Authors’ contributions

MTAD and CCM assembled datasets. CCM, MTAD and AMM identified SGOs, aligned and quality checked all multiple sequence alignments. CCM carried out all phylogenetic, recombination, human population and statistical analyses. CCM, MTAD and NBL carried out selective pressure analyses. CCM and AMM carried out functional studies on data. CCM and MJO’C conceived of the study, its design and coordination. CCM, AMM, MTAD, ECT, CS, NBL, and MJO’C, contributed to drafting the manuscript. All authors read and approved the final manuscript.

## Supplementary Material

Additional file 1 **A summary of the telomere associated gene data used in the analysis.** A list of genes and their associated role in telomere maintenance are given, followed by the number of sequences present in each species, the length (in base pairs) of the alignment and the following cells indicate the number of times a sequence is observed in a specific species. Click here for file

Additional file 2 **Divergence results using CladeC for each gene family.** The lineage tested, the estimated CladeC lnL value, M2_Rel lnL value, the p-value, the adjusted p-value for multiple testing (p-adj) and estimated parameters are given for the lineage-specific analysis for each of the genes analysed. Click here for file

Additional file 3 **Lineage-specific CodeML (modelA) results for each gene family.** The lineage tested, the estimated modelA, modelAnull and M1Neutral lnL values are given followed by the p-value and p-adj value for multiple testing for modelA vs modelAnull LRT and modelA vs model1Neutral LRT. A list of BEB positively selected sites with a PP > 0.90 are given for lineage-specific analysis for each of the genes analysed. Click here for file

Additional file 4 **Results for estimates of saturation at synonymous sites.** The gene name is given in the first column. The result of the Ds saturation test is given in the second column, where a Ds substitution per site exceeding 2 on any given branch implies saturation. The phylogenetic tree estimated from a distance matrix of Ds sites is given in the last column. Click here for file

Additional file 5 **Results of Recombination Analysis.** For each gene the region where recombination was detected in each species is given. The minor and major donor parental sequences are shown, followed by support values for each of the recombination detection methods used. NS = No Significant P-value recorded. Click here for file

Additional file 6 **Details of the genomes used in the study.** For each species the common name and latin name are given along with the genome version used from the Ensembl database and the associated species codes. Click here for file

Additional file 7 Alignment Data for each gene families analysed. Click here for file

Additional file 8 Gene Trees for each gene family analysed. Click here for file
